# Supplementary material for: Elevated Plasma D-Dimer Concentrations in Adults after an Outpatient-Treated COVID-19 Infection
Source: Viruses. 2022 Nov 3;14(11):2441. doi: 10.3390/v14112441 (PMC9699049; doi:10.3390/v14112441)
Supplement: Supplementary file 1 [file viruses-14-02441-s001.zip › viruses-1945435-supplementary.pdf]

Elevated Plasma D-Dimer concentrations in Adults after An Outpatient-Treated COVID-19 Infection

Christa Meisinger, Inge Kirchberger, Tobias D Warm, Alexander Hyhlik-Dürr, Yvonne Goßlau and Jakob Linseisen

Supplementary material

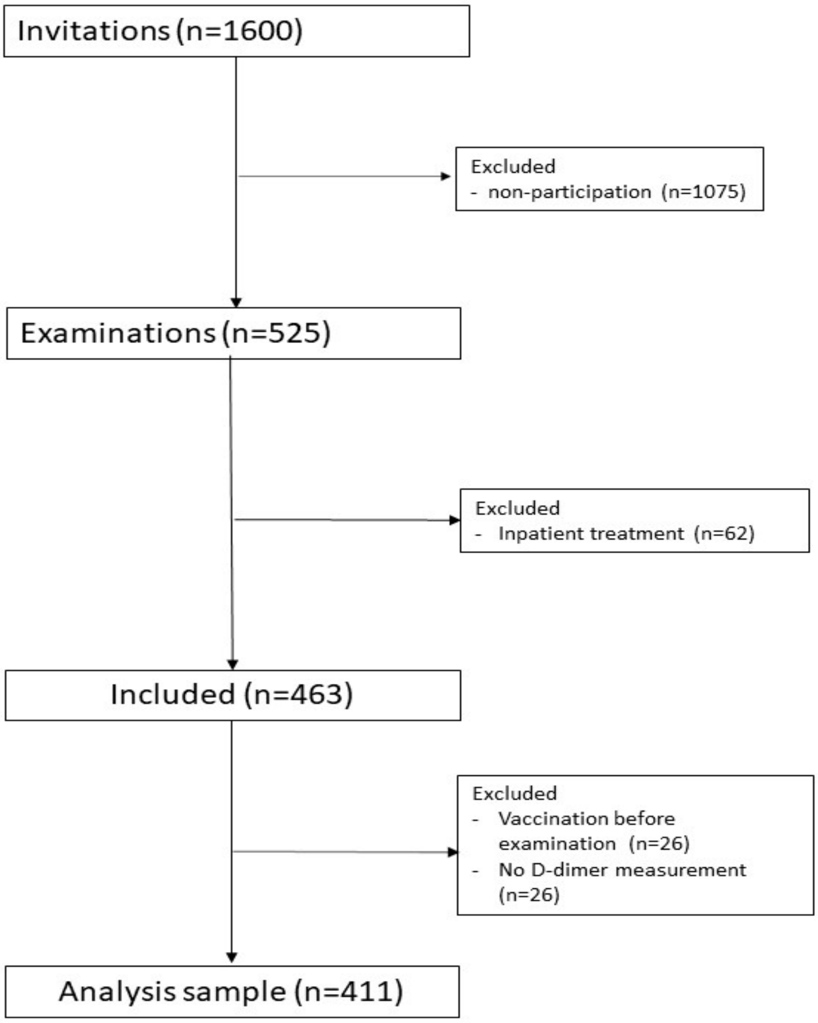

Figure S1. Flow chart.

Table S1. Characteristics of post-COVID-19 patients treated as outpatients (given as frequencies and percentages, or median and interquartile range), by plasma D-dimer concentrations of >500 to <1000 and >= 1000 µg/L.

	D-dimer >500 to <1000 µg/L	D-dimer ≥1000 µg/L	p-value*
**Clinical characteristics**	n = 44	n = 17	
Sex (males)	16 (36.4)	7 (41.2)	0.7737
Age (years)	57 (48.5;66.5)	64 (52;73)	0.1224
School education (high)	29 (65.9)	11 (64.7)	1.000
Marital status (yes)	36 (81.8)	11 (64.7)	0.1838
Body mass index (kg/m^2)	25.9 (22.7; 29.1)	24.8 (22.5; 27.3)	0.3807
Hypertension (yes)	13 (29.6)	6 (35.3)	0.7605
Diabetes mellitus (yes)	3 (6.8)	1 (5.9)	1.000
Depression (yes)	3 (6.8)	2 (11.8)	0.6121
Cardiovascular disease (yes)	5 (11.4)	3 (17.7)	0.6740
Venous thromboembolism before infection (yes)	2 (4.6)	4 (23.5)	0.0457
Chronic venous insufficiency (yes)	10 (22.7)	4 (23.5)	1.000
Current smoker (yes)	18 (40.9)	10 (58.8)	0.2581
Post COVID-19 fatigue (yes)	17 (38.6)	5 (29.4)	0.5651
Time since acute infection (days)	246.5 (135;322)	260 (158;359)	0.6039
Anticoagulation therapy (yes)	2 (4.6)	1 (5.9)	1.000
**Laboratory parameters**			
White blood cell count (/nl)	7.15 (6.2;7.74)	7.50 (5.63;8.57)	0.5095
Hemoglobin (g/L)	140.5 (132.5;145)	139 (126;148)	0.4686
Platelets (/nl)	220.5 (207.5;265)	260 (189;281)	0.6466
Glucose (mg/dL)	87.5 (80.5;108)	96 (79;114)	0.6123
aPTT (sec)	28 (26;31)	29 (25;31)	0.9548
C-reactive protein (mg/dL)	0.12 (0.06;0.37)	0.16 (0.08;0.31)	0.5819
IL-6 (pg/ml) [a]	3.50 (2.50; 3.815)	3.62 (3.50; 4.14)	0.0610
D-dimer (µg/L)	654 (562; 740)	1475 (1143; 2384)	<.0001
Anti-β2-glycoprotein IgG antibodies (U/ml)	2.35 (2.0;3.5)	2.4 (2.0;4.6)	0.6504
Anti-β2-glycoprotein IgM antibodies (U/ml)	5.55 (2.0;11.75)	5.2 (2.9;8.3)	0.7888
Anticardiolipin IgG antibodies (U/ml)	2.0 (2.0;2.1)	2.0 (2.0;2.8)	0.4948
Anticardiolipin IgM antibodies (U/ml)	2.0 (2.0;3.3)	2.0 (2.0;2.1)	0.5451
SARS-CoV-2 IgG antibodies (U/ml)	130.5 (44.5;267.5)	327 (94.5;726)	0.0943

[a]based on 40 and 13 participants, respectively; *Fisher's exact test or Wilcoxon Rank Sum Test
